# Mini-FLOTAC as an alternative, non-invasive diagnostic tool for *Schistosoma mansoni* and other trematode infections in wildlife reservoirs

**DOI:** 10.1186/s13071-019-3613-6

**Published:** 2019-09-16

**Authors:** Stefano Catalano, Amelia Symeou, Kirsty J. Marsh, Anna Borlase, Elsa Léger, Cheikh B. Fall, Mariama Sène, Nicolas D. Diouf, Davide Ianniello, Giuseppe Cringoli, Laura Rinaldi, Khalilou Bâ, Joanne P. Webster

**Affiliations:** 1 Centre for Emerging, Endemic and Exotic Diseases, Department of Pathobiology and Population Sciences, The Royal Veterinary College, University of London, Hatfield, AL97TA UK; 2 London Centre for Neglected Tropical Disease Research, School of Public Health, Faculty of Medicine, Imperial College London, London, W21PG UK; 3 Faculté de Médecine, de Pharmacie et d’Odonto-Stomatologie, Université Cheikh Anta Diop, BP 5005, Dakar, Senegal; 4 Unité de Formation et de Recherche des Sciences Agronomiques, de l’Aquaculture et des Technologies Alimentaires, Université Gaston Berger, BP 234, Saint-Louis, Senegal; 5 Department of Veterinary Medicine and Animal Production, University of Naples Federico II, 80137 Naples, Italy; 6 Centre de Biologie et de Gestion des Populations, Institut de Recherche pour le Développement, BP 1386, Dakar, Senegal

**Keywords:** Diagnostics, *Mastomys huberti*, Mini-FLOTAC, Parasites, Schistosomiasis, Trematodes, Wildlife, West Africa, Zoonoses

## Abstract

**Background:**

Schistosomiasis and food-borne trematodiases are not only of major public health concern, but can also have profound implications for livestock production and wildlife conservation. The zoonotic, multi-host nature of many digenean trematodes is a significant challenge for disease control programmes in endemic areas. However, our understanding of the epidemiological role that animal reservoirs, particularly wild hosts, may play in the transmission of zoonotic trematodiases suffers a dearth of information, with few, if any, standardised, reliable diagnostic tests available. We combined qualitative and quantitative data derived from post-mortem examinations, coprological analyses using the Mini-FLOTAC technique, and molecular tools to assess parasite community composition and the validity of non-invasive methods to detect trematode infections in 89 wild Hubert’s multimammate mice (*Mastomys huberti*) from northern Senegal.

**Results:**

Parasites isolated at post-mortem examination were identified as *Plagiorchis* sp., *Anchitrema* sp., *Echinostoma caproni*, *Schistosoma mansoni*, and a hybrid between *Schistosoma haematobium* and *Schistosoma bovis*. The reports of *E. caproni* and *Anchitrema* sp. represent the first molecularly confirmed identifications for these trematodes in definitive hosts of sub-Saharan Africa. Comparison of prevalence estimates derived from parasitological analysis at post-mortem examination and Mini-FLOTAC analysis showed non-significant differences indicating comparable results between the two techniques (*P* = 1.00 for *S. mansoni*; *P* = 0.85 for *E. caproni*; *P* = 0.83 for *Plagiorchis* sp.). A Bayesian model, applied to estimate the sensitivities of the two tests for the diagnosis of *Schistosoma* infections, indicated similar median posterior probabilities of 83.1% for Mini-FLOTAC technique and 82.9% for post-mortem examination (95% Bayesian credible intervals of 64.0–94.6% and 63.7–94.7%, respectively).

**Conclusions:**

Our results showed that the Mini-FLOTAC could be applied as an alternative diagnostic technique for the detection of the zoonotic *S. mansoni* and other trematodes in rodent reservoirs. The implementation of non-invasive diagnostics in wildlife would offer numerous advantages over lethal sampling methodologies, with potential impact on control strategies of zoonotic helminthiases in endemic areas of sub-Saharan Africa and on fostering a framework of animal use reduction in scientific practice.

## Background

Digenean trematodes (phylum Platyhelminthes) are characterised by complex life cycles involving replication by asexual reproduction within their intermediate hosts and transmission to vertebrate definitive hosts *via* ingestion, with sexual reproduction of the hermaphroditic adult parasites in their final infection site [1]. The sole exception is represented by members of the family Schistosomatidae, which are dioecious parasites (i.e. separate sexes) infecting their definitive host *via* skin penetration [2]. Trematodiases are of great medical and veterinary importance, responsible for public health issues, economic losses, and conservation concerns [3–6]. Estimates from the World Health Organization show that globally about 220 million people required preventive treatment for schistosomiasis in 2017 [7], while millions of people are suffering one or more food-borne trematodiases [8].

The complex multi-host, zoonotic nature of trematodiases may have a considerable impact on the outcome of disease control programmes in endemic areas [9, 10]. The role of wild small mammals as disease reservoirs is emerging as a public health concern, and the involvement of rodents in the transmission of human agents of schistosomiasis (e.g. *Schistosoma japonicum* and *Schistosoma mansoni*) in different regions of the world is a noteworthy example [11–13]. To date, the characterisation of helminth communities infecting wildlife has largely relied on lethal sampling, severely restricting the host species that can be studied, the adequacy of sampling strategies and sizes, and the scope of the scientific questions that can be addressed [14]. However, diagnostic approaches based on faecal egg count (FEC) techniques alone in wild hosts are inevitably limited to a coarse morphological identification of parasitic elements, often to the taxonomic ranks of either order or family, preventing the fine-scale partition of parasite fauna composition [14, 15]. DNA-based methods could contribute significantly to the correct identification of parasitic taxa while implementing non-invasive sampling strategies. Nevertheless, the exclusive application of molecular techniques may under represent parasite community composition and inaccurately depict quantitative estimates of infection if inferences are not properly tested [9, 14].

The diagnostic accuracy and applicability of a range of methodologies have been tested for the improved detection of trematodiases in humans (e.g. rapid tests for circulating antigens, urine and stool microscopy, serological tests, and DNA-based methods) [16–18]. In contrast, our understanding of the epidemiological role that animal reservoirs, particularly wild hosts, may play in the transmission of zoonotic trematodiases is constrained by a dearth of information and standardised, reliable diagnostic tests available [9]. Our aim was to assess trematode infections in wild Hubert’s multimammate mice (*Mastomys huberti*) from northern Senegal *via* the concerted application of post-mortem examination, FEC using the Mini-FLOTAC technique, and molecular analysis. Mini-FLOTAC, combined with Fill-FLOTAC, is a tool based on the flotation of parasitic eggs without requiring a centrifuge (and therefore power supply) for processing [19]. Furthermore, a portion of the faecal samples can be fixed in formalin and stored prior to the analysis, making the method versatile and easy to implement in resource-limited field settings [19–21]. In particular, our objective was to assess the performance of the Mini-FLOTAC as an alternative tool for the detection of *Schistosoma* infections in rodent reservoirs, and therefore its future applicability within non-invasive sampling schemes.

## Methods

### Post-mortem examination

Between May 2016 and December 2017, sampling of small mammals was conducted at sites in and around the town of Richard Toll (16° 27ʹ N, 15° 41ʹ W) and on the shores of Lake Guiers (16° 15ʹ N, 15° 51ʹ W), Senegal, following methodologies previously described [13, 22]. At post-mortem examination of *M. huberti*, thoracic and abdominal organs were dissected, scraped, washed with tap water, and observed for the presence of helminths using a glass tray against a black background. The isolated adult digeneans were microscopically identified to the genus level based on their morphology (see identification keys in [23]), counted to quantify infection intensity, and stored in 95% ethanol at − 20 °C until molecular analysis. Morphological identification of preserved specimens was obtained after staining in Semichon’s carmine, immersion in clearing medium (i.e. ethanol followed by xylene), and mounting on a microscope slide using Canada balsam. For *Plagiorchis* isolates, infection intensity was quantified up to 61 worms per organ; time constraints during fieldwork prevented the integral count of *Plagiorchis* parasites observed in the biliary tract and/or small intestine, therefore the value > 61 was used to indicate higher intensities. During post-mortem examinations, faecal material from the necropsied individuals was collected from the rectum (*n* = 89) and from underneath the wire-mesh live trap (*n* = 8) into separate vials, weighted (0.1–0.7 g), and stored in 1.5 ml of 10% neutral-buffered formalin.

### Molecular analysis

After rehydration in nuclease-free water, DNA from individual trematode specimens was extracted using either the Epicentre® MasterPure™ Complete DNA and RNA Purification Kit (Epicentre Biotechnologies, Madison, WI, USA) or the Qiagen DNeasy^®^ Blood & Tissue Kit (Qiagen, Hilden, Germany) following the manufacturer’s instructions. DNA extracts were eluted in 30 μl TE buffer and amplified for the internal transcribed spacer (ITS) of the nuclear ribosomal DNA (rDNA) and the partial cytochrome *c* oxidase subunit 1 gene (*cox*1) of the mitochondrial DNA (mtDNA) using the primer pairs ETTS1 (5′-TGC TTA AGT TCA GCG GGT-3′) and ETTS2 (5′-AAC AAG GTT TCC GTA GGT GAA-3′) [24], and 2575 (5′-TTT TTT GGG CAT CCT GAG GTT TAT-3′) and 3021 (5′-TAA AGA AAG AAC ATA ATG AAA ATG-3′) [25], respectively. Enzymatic amplification for polymerase chain reaction (PCR) was performed in 25 µl reaction mixtures including PuReTaq^™^ Ready-To-Go^™^ PCR Beads (GE Healthcare UK Limited, Little Chalfont, UK), 0.5 µmol/l of each primer and 2 µl DNA template. Cycling parameters for the ITS region consisted of an initial nucleic acid denaturation at 95 °C for 5 min, followed by 35 cycles of 95 °C for 30 s, 56 °C for 1 min, and 72 °C for 1 min, with a final extension step for 7 min at 72 °C. Cycling parameters for the *cox*1 gene consisted of an initial nucleic acid denaturation at 94 °C for 5 min, followed by 35 cycles of 94 °C for 30 s, 52 °C for 1 min, and 72 °C for 1 min, with a final extension step for 7 min at 72 °C. PCR products were sequenced using the original PCR primers in a 3730xl DNA Analyzer system by Eurofins Genomics (Ebersberg, Germany). Contig assembly and editing were performed with CodonCode Aligner v8.0.1 (CodonCode Corporation, Centerville, MA, USA) and the resulting sequences were compared by alignment with data available in the GenBank database.

### Mini-FLOTAC technique

Faecal samples were analysed between four and six months after their collection date using the Fill-FLOTAC 2 and Mini-FLOTAC devices [19], together with a flotation solution (FS) made of zinc sulphate heptahydrate (H_14_O_11_SZn) and tap water (FS7, see [26] for further details on the different FS types). This FS7 was confirmed to be at a density of 1.35 with a hydrometer (Brannan, Cleator Moor, UK). Each faecal sample was fully transferred into a Fill-FLOTAC 2, 13.5 ml of FS7 were added to reach 1:10 dilution ratio, and the specimen was homogenised in order to fill the Mini-FLOTAC chambers following standard operating procedures [19]. After an average waiting time of 10 min to allow the flotation of parasitic eggs, we performed a double-blind observation of both Mini-FLOTAC ruled grids under an Olympus CX41 microscope equipped with an Olympus DP20 camera, counting all the parasitic eggs we observed. Eggs per gram (EPG) estimates, herein considered a proxy for infection intensity, were calculated following the described protocol [19]: we multiplied the obtained number of parasitic eggs by the multiplication factor, which was derived from dividing the dilution factor by the analysed volume (i.e. 2 ml) in the Mini-FLOTAC chambers (Table 1).

**Table 1:** Grams of faeces, dilution factors, and multiplication factors used to calculate eggs per gram estimates derived from the trematode egg counts

Grams of faeces	Dilution factor^a^	Multiplication factor^b^
0.1	150.0	75.0
0.2	75.0	37.5
0.3	50.0	25.0
0.4	37.5	18.5
0.5	30.0	15.0
0.6	25.0	12.5
0.7	21.5	10.5

^a^Calculated by dividing the final volume of the solution (15 ml) by the amount of faeces analysed (0.1–0.7 g)

^b^Calculated by dividing the corresponding dilution factor by the analysed volume of 2 ml

### Statistical analysis

Statistically significant differences in the proportion of positive individuals were analysed using Pearson’s chi-square test. After the data distribution was assessed as non-normal, significant correlations in the intensity of trematode infections between post-mortem and faecal examinations were analysed using the non-parametric Spearman’s rank correlation (*ρ*) coefficient. Confidence intervals (CI) at 95% level were calculated for proportions of positive individuals using the Agresti-Coull interval [27]. Statistical tests, considered significant when *P* ≤ 0.05, were implemented in R v3.1.2.

A Bayesian model was applied to estimate the adjusted (true) proportion of individuals positive to *Schistosoma* infection and the diagnostic accuracy of post-mortem examination and Mini-FLOTAC technique. The model was based on the assumption that the probability (*p*) of a positive test for each technique can be expressed as *p* = *π Se* + (1 − *π*) (1 − *Sp*), where *π* represents the true proportion of infection in the population, while *Se* and *Sp* represent the sensitivity and specificity of the diagnostic techniques, respectively [28]. Prior estimates of the sensitivity (i.e. the proportion of true positives that are correctly identified as such) for post-mortem examination and Mini-FLOTAC technique could not be derived since data, applicable to the surveyed host population, were not available. We used uninformative *β*-distribution priors (*β* ~ (1, 1)), equivalent to a uniform distribution ranging from zero to one. Specificity (i.e. the proportion of true negatives that are correctly identified as such) of each test was assumed to be 100%. Posterior probabilities were inferred using JAGS v4.3.0 [29] in conjunction with R v3.5.1 (through the *rjags* and *coda* packages), implementing two Markov Chain Monte Carlo chains, 200,000 iterations, ‛burn-inʼ of 5000, and thinning interval of 40.

## Results

Based on the combined morphological and molecular analysis of rDNA and mtDNA data, the trematodes collected at post-mortem were identified as *Echinostoma caproni*, *Plagiorchis* sp., *Anchitrema* sp., *S. mansoni*, and a hybrid between *Schistosoma haematobium* and *Schistosoma bovis*. Overall, these parasites were isolated in 86 out of 89 *M. huberti* (96.6%; 95% CI: 90.6–98.8%), with: *Plagiorchis* sp. in the biliary tract and/or small intestine of 78 hosts (87.6%; 95% CI: 79.0–93.1%); *Schistosoma* spp. in the portal system and/or mesenteric vessels of 21 hosts (23.6%; 95% CI: 15.9–33.5%); *E. caproni* in the hepatic parenchyma or small intestine of 15 hosts (16.9%; 95% CI: 10.4–26.1%); and *Anchitrema* sp. in the small intestine of three hosts (3.4%; 95% CI: 0.7–9.9%).

**Fig. 1:**
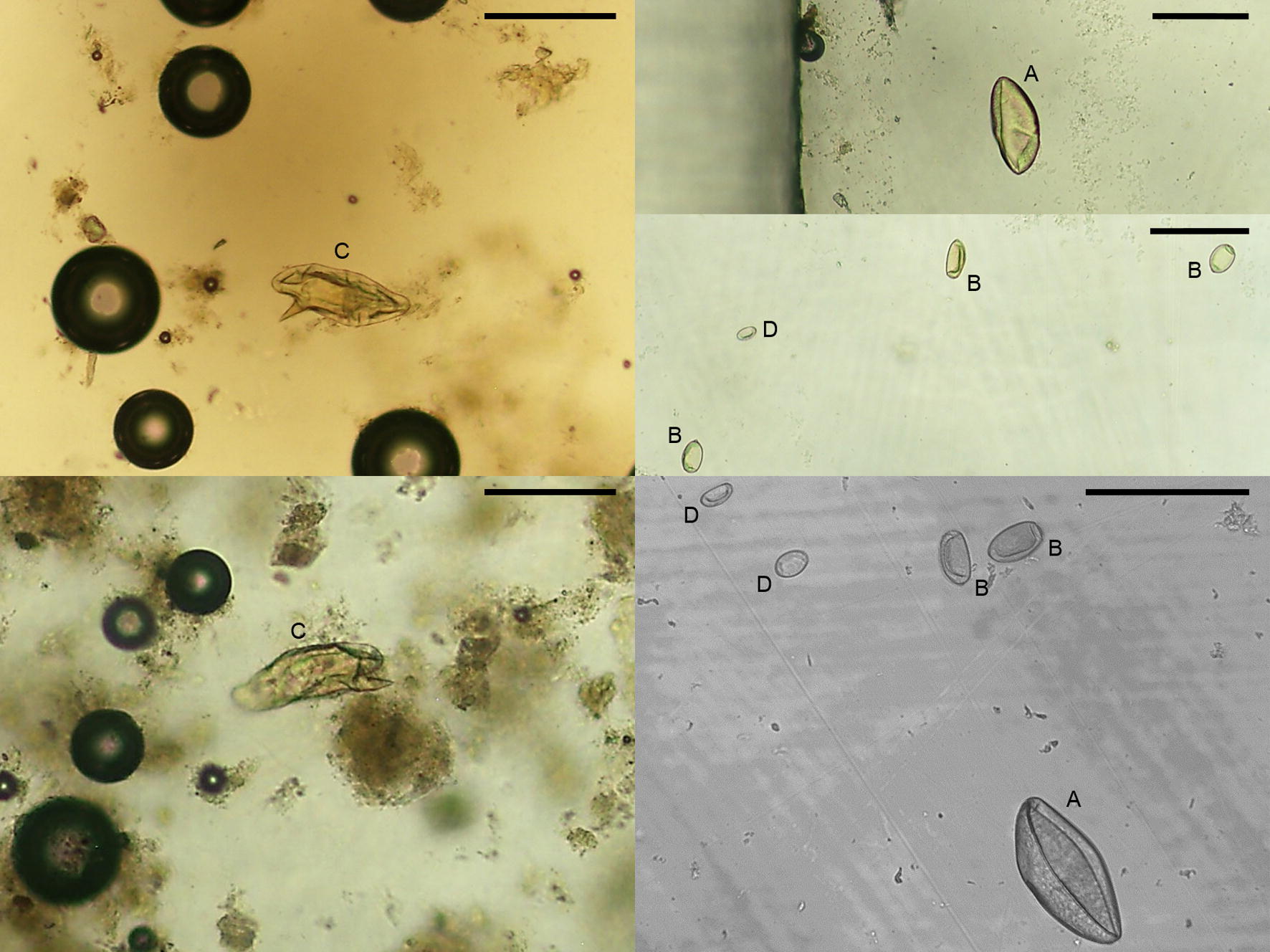
Eggs of *Echinostoma caproni* (A), *Plagiorchis* sp. (B), *Schistosoma mansoni* (C), and *Anchitrema* sp. (D) observed under the microscope during Mini-FLOTAC analysis (scale bars: 100 μm)

The Mini-FLOTAC analysis identified parasitic eggs in 85 out of 89 individuals (95.5%; 95% CI: 88.7–98.6%), which were morphologically compatible with: *Plagiorchis* sp. in 76 hosts (85.4%; 95% CI: 76.5–91.4%); *S. mansoni* in 21 hosts (23.6%; 95% CI: 15.9–33.5%); *Echinostoma* sp. in 18 hosts (20.2%; 95% CI: 13.1–29.8%); and *Anchitrema* sp. in one host (1.1%; 95% CI: 0–0.7%) (Fig. 1). Results of the combined post-mortem examination, molecular analysis, and Mini-FLOTAC technique are summarised in Table 2. Remarkably, three hosts were positive to *S. mansoni* during the Mini-FLOTAC analysis while their post-mortem examination was negative and *vice versa*; for *E. caproni*, three hosts were negative at post-mortem whereas their Mini-FLOTAC analysis resulted positive.

**Table 2:** Percent prevalence (95% confidence intervals in parentheses) and intensity (median and range in parentheses) of trematode infections in Hubert’s multimammate mice (*Mastomys huberti*) at post-mortem examination (infection intensity expressed as parasite counts) and Mini-FLOTAC analysis (infection intensity expressed as eggs per gram)

Parasite	Infection	Adults (*n* = 80)		Juveniles (*n* = 9)		Total (*n* = 89)
		Males (*n* = 45)	Females (*n* = 35)	Males (*n* = 3)	Females (*n* = 6)	
*Plagiorchis* sp.						
Post-mortem	Prevalence (%)	86.7 (73.4–94.1)	85.7 (70.1–94.2)	100 (38.3–100)	100 (55.7–100)	87.6 (79.0–93.1)
	Intensity	16 (1– > 61)	26.5 (2– > 61)	19 (3–45)	21.5 (3– > 61)	18 (1– > 61)
Mini-FLOTAC	Prevalence (%)	84.4 (70.9–92.6)	85.7 (70.1–94.2)	66.7 (20.2–94.4)	100 (55.7–100)	85.4 (76.5–91.4)
	Intensity	4687.5 (92–123,675)	4,350 (25–134,900)	2,962.5 (1725–2,475)	5,212 (610–91,125)	4,300 (25–134,900)
*Schistosoma mansoni*						
Post-mortem	Prevalence (%)	24.4 (14.1–38.8)	25.7 (14.0–42.3)	–	16.7 (1.1–58.2)	23.6 (15.9–33.5)
	Intensity	5 (20–35)	17 (2–64)^a^	–	2 (na)	8 (2–64^a^)
Mini-FLOTAC	Prevalence (%)	22.2 (12.3–36.5)	28.6 (16.2–45.2)	–	16.7 (1.1–58.2)	23.6 (15.9–33.5)
	Intensity	293.5 (50–1237)	137.5 (15–900)	–	600 (na)	262 (15–1237)
*Echinostoma caproni*						
Post-mortem	Prevalence (%)	20.0 (10.7–34.0)	14.3 (5.8–29.9)	33.3 (5.6–79.8)	–	16.9 (10.4–26.1)
	Intensity	2 (1–32)	5 (1–34)	1 (na)	–	2 (1–34)
Mini-FLOTAC	Prevalence (%)	26.7 (15.8–41.2)	14.3 (5.8–29.9)	33.3 (5.6–79.8)	–	20.2 (13.1–29.8)
	Intensity	135 (18–52,275)	315 (37–1,375)	675 (na)	–	232 (18–52,275)
*Anchitrema* sp.						
Post-mortem	Prevalence (%)	2.2 (0–12.6)	5.7 (0.6–19.6)	–	–	3.4 (0.7–9.9)
	Intensity	9 (na)	12.5 (1–24)	–	–	9 (1–24)
Mini-FLOTAC	Prevalence (%)	–	2.9 (0–15.8)	–	–	1.1 (0–0.7)
	Intensity	–	750 (na)	–	–	750 (na)

^a^One pair was formed of *S. mansoni* male and *S. haematobium*/*S. bovis* hybrid female [13]; however, only eggs with lateral spine, identified as *S. mansoni* eggs, were observed

*Abbreviation*: na, not applicable

The proportion of individuals positive for trematodes was not significantly different between post-mortem examination and Mini-FLOTAC technique, neither when *Plagiorchis* sp. (*χ*^2^ = 0.05, *df* = 1, *P* = 0.83), *S. mansoni* (*χ*^2^ = 0.00, *df* = 1, *P* = 1.00), and *E. caproni* (*χ*^2^ = 0.04, *df* = 1, *P* = 0.85) were considered singularly, nor when they were grouped (*χ*^2^ = 0.15, *df* = 1; *P* = 0.70). The median posterior estimate of the adjusted (true) proportion of individuals positive to *Schistosoma* infection obtained by the Bayesian model in the surveyed *M. huberti* was 28.3% (95% Bayesian credible intervals of 19.4–38.5%). Median posterior sensitivity estimates for Mini-FLOTAC technique and post-mortem examination in the diagnosis of *Schistosoma* infections were 83.1% (95% Bayesian credible intervals of 64.0–94.6%) and 82.9% (95% Bayesian credible intervals of 63.7–94.7%), respectively. Correlation between infection intensities at post-mortem examination (i.e. adult parasite counts) and Mini-FLOTAC analysis (i.e. EPG) was not significant for *Plagiorchis* sp. (*ρ* = 0.18, *P* = 0.19), whereas it was weak for both *E. caproni* (*ρ* = 0.51, *P* = 0.053) and *S. mansoni* (when considering the number of schistosome pairs *ρ* = 0.43, *P* = 0.038; when considering the number of schistosome individuals *ρ* = 0.46, *P* = 0.058). Comparisons for *Anchitrema* sp. could not be made given the small number of infections observed. Similarly, the eight faecal specimens collected from underneath the wire-mesh live trap were not sufficient for meaningful statistical comparisons; however, sensitivity of the Mini-FLOTAC technique was identical to the results obtained on rectal faecal samples collected from the same individual (Table 3).

**Table 3:** Trematode infection intensity at post-mortem examination (PME) and Mini-FLOTAC analysis (MF), expressed as parasite counts and eggs per gram, respectively, of eight Hubert’s multimammate mice (*Mastomys huberti*) for which faecal samples were collected from both the rectum and underneath the trap. The weight of the analysed faecal material from the rectum and the trap (in parentheses) is reported

Host ID	Weight of faeces (g)	Parasite	PME	MF rectum	MF trap
SC372	0.2 (0.1)	*Plagiorchis* sp.	2	1200	2700
SC388	0.4 (0.1)	*Plagiorchis* sp.	7	1831	8250
SC404	0.2 (0.1)	*Plagiorchis* sp.	3	–	–
SC417	0.3 (0.1)	*Plagiorchis* sp.	26	700	600
SC491	0.5 (0.3)	*Plagiorchis* sp.	> 61	10,350	31,600
		*Schistosoma mansoni*	–	15	50
SC492	0.2 (0.1)	*Plagiorchis* sp.	4	84,112	57,975
		*Schistosoma mansoni*	35^a^	1237	3225
SC496	0.2 (0.1)	*Plagiorchis* sp.	> 61	31,875	34,950
		*Schistosoma mansoni*	18	337	675
SC504	0.1 (0.1)	*Plagiorchis* sp.	3	39,750	10,050

^a^One pair was formed of *S. mansoni* male and *S. haematobium*/*S. bovis* hybrid female [13]; however, only eggs with lateral spine, identified as *S. mansoni* eggs, were observed

For all trematode species, representative specimens were archived at the Natural History Museum (London, UK) under the accession numbers 2018.3.7.33-38 (*E. caproni*), 2018.3.7.39-67 (*Plagiorchis* sp.), and 2019.2.13.1-3 (*Anchitrema* sp.). Schistosome trematodes were stored in the Schistosomiasis Collection at the Natural History Museum (SCAN) [30]. Sequencing data were deposited in the GenBank database for *Schistosoma* spp. (accession numbers MF776585-97 for ITS and MF919405-28 for *cox*1) [13], *Plagiorchis* sp. (accession numbers MH633855-62 for ITS and MH673675-82 for *cox*1) [22], and *E. caproni* (accession numbers MK721181-2 for ITS and MK732350-1 for *cox*1).

## Discussion

The combination of post-mortem examination, Mini-FLOTAC, and molecular analysis was used to assess parasite community composition and FEC as a valid diagnostic method to investigate rodents as reservoirs of zoonotic and non-zoonotic trematodes. The results showed comparable sensitivity estimates for parasitological examination at post-mortem and Mini-FLOTAC, suggesting that this FEC technique could be further implemented in non-invasive sampling strategies targeting trematode infections in rodents. In particular, the diagnostic sensitivity to *S. mansoni* infections identified the Mini-FLOTAC as a reliable tool for future surveys on rodent reservoirs in the many regions where schistosomiasis is endemic, with the potential to significantly reduce the use of lethal sampling methods. Individuals that were positive for *S. mansoni* and *E. caproni* at FEC while negative at post-mortem examination, and *vice versa*, highlighted the absence of a gold standard diagnostic test potentially due to flaws inherent to either technique [31], their application by the operator [32], and the dynamics of parasitic infections (e.g. see [33] on overlooked single-sex *Schistosoma* spp. infections). Furthermore, the FS7 used herein for the flotation of trematode eggs interacted and partially altered the classical morphology of those parasitic elements, which suggests that calibration trials and training may be required before using the Mini-FLOTAC device [20, 34]. Recent studies have developed non-invasive approaches to combine and compare qualitative/quantitative data derived from FEC and molecular identification of parasitic taxa in wildlife [14, 35, 36]. These efforts should draw attention to the lack of tested diagnostic tools available for animal reservoirs of zoonotic helminthiases [9] and prompt the development of standardised techniques. These could be further implemented within non-lethal sampling schemes as outlined by the principles of replacement, refinement, and reduction of animal use in wildlife research (https://www.nc3rs.org.uk/wildlife-research). Such tools could offer numerous advantages over destructive methodologies in epidemiological studies, including access to larger sample sizes and repeated sampling of individuals/populations in order to explore longitudinal changes and other aspects of infection dynamics [14].

During our study, statistical correlations between infection intensity at post-mortem examination and EPG using the Mini-FLOTAC technique resulted weak for both *S. mansoni* and *E. caproni*. The accuracy of the Mini-FLOTAC is correlated with the amount of faeces examined [19]. However, while EPG values may have been affected by the inflated dilution and multiplication factors as a consequence of the small amount of faecal material analysed (i.e. between 0.1 and 0.7 g), our estimates were similar to those obtained during experimental infections of *M. huberti* with *S. mansoni* [37]. These findings support the high infection intensity values, and potentially contamination index (i.e. daily faecal excretion rates of *S. mansoni* eggs), for *M. huberti*, as also observed during epidemiological surveys of rodents harbouring *S. mansoni* in Brazil [38] and *S. japonicum* in China [39]. A further limitation may have been the long storage time in 10% formalin before processing of the specimens (i.e. between four and six months after collection date). Previous studies and the published protocol for the Mini-FLOTAC advise storage in 5% formalin and for a maximum of approximately one month in order to not impair the sensitivity of the technique [19, 21]. However, our study showed that the diagnostic sensitivity to *S. mansoni* did not appear to be affected by a longer waiting time before analysis.

The combined morphological and molecular analyses enabled the unequivocal identification of the digenean trematodes isolated at post-mortem examination. The finding of *S. mansoni* and *S. haematobium/S. bovis* hybrid has been previously discussed [13]. Similarly, the epidemiology, pathology, and molecular systematics of *Plagiorchis* sp., a previously undescribed West African lineage, has been documented [22]. To our knowledge, the reports of *E. caproni* and *Anchitrema* sp., described herein, are the first molecularly confirmed identifications for these trematodes in definitive hosts of sub-Saharan Africa (see [40–42] for data on gastropod intermediate hosts of *E. caproni* in the African continent). The life cycle of *Anchitrema* parasites is largely undetermined since, to date, they have only been recorded in the intestine of various definitive hosts in the tropics and subtropics [23, 43]. *Anchitrema sanguineum* is the most frequently reported taxon in mammalian hosts but, to date, identifications have been solely based on morphological traits. This species has been sporadically isolated from rats in Egypt [44] and Thailand [45], from the bat *Myotis velifer* in Mexico [46], and even from the intestinal tract of a domestic dog and a human in Thailand, whose infection was hypothesised to have occurred *via* oral transmission [43]. In contrast, the biology of *E. caproni* and other *Echinostoma* spp. is well studied. These parasites use freshwater gastropods, primarily of the families Planorbidae (e.g. *Biomphalaria* and *Bulinus* spp.) and Lymnaeidae, as first intermediate hosts, while fish, molluscs, crustaceans, and amphibians can serve as second intermediate hosts. Infection of definitive hosts (i.e. birds and mammals, including humans), and development into hermaphroditic adult parasites in their intestine and/or biliary tract, occurs by ingestion of metacercariae harboured by the second intermediate host [41, 47–49]. Therefore, echinostomiasis is considered a food-borne zoonosis: whilst the disease does not usually show clinical signs when the infection burden is low, gastrointestinal symptoms and pronounced weight loss can occur with severe infections [47, 50]. Rodents may act as reservoirs of zoonotic *Echinostoma* spp. by perpetuating the contamination of freshwater bodies with the parasitic eggs *via* defecation [51, 52]. However, identification of *Echinostoma* spp. and differentiation between zoonotic and non-zoonotic species can be difficult without a molecular approach due to the morphological similarity among members within the genus [53, 54].

## Conclusions

We combined classical and molecular parasitological analyses for species identification and diagnostics testing of zoonotic and non-zoonotic trematodes of wildlife. Our results indicated that the Mini-FLOTAC represents a reliable technique to detect the zoonotic *S. mansoni* and other parasites in rodent reservoirs. A growing body of information on helminth communities of West African rodents is gradually enhancing our understanding of host use and transmission dynamics [55–57]. To date, the quantification of adult helminths during post-mortem examination remains the gold standard technique for assessing infection in wildlife; as a consequence, non-invasive methods are often untested or sporadically applied [9, 14]. Therefore, additional studies will be necessary to implement the use of the Mini-FLOTAC in non-invasive sampling strategies targeting animal hosts. Future advancements should incorporate FEC diagnostics when obtaining baseline data while testing coprological DNA-based methods. This approach would contribute significantly towards a higher diagnostic throughput and a deeper understanding of the interactions between a parasite and its host community, with potential impact on control strategies of zoonotic helminthiases and, ultimately, on fostering a framework of animal use reduction in scientific practice.

## Data Availability

All data generated or analysed during this study are included in this published article. Parasite specimens and sequencing data from this study are available in the collection of the Natural History Museum (London, UK) and in the GenBank database, respectively, under the accession numbers listed in this manuscript.
